# Preoperative prolonged fasting causes severe metabolic acidosis: A case report

**DOI:** 10.1097/MD.0000000000017434

**Published:** 2019-10-11

**Authors:** WenQin Zhou, LinLi Luo

**Affiliations:** a Department of Anesthesiology, West China Second University Hospital, Sichuan University; b Key Laboratory of Birth Defects and Related Diseases of Women and Children, Ministry of Education (Sichuan University), Chengdu, China.

**Keywords:** enhanced recovery after surgery, metabolic acidosis, prolonged fasting

## Abstract

**Rationale:**

Preoperative prolonged fasting may cause starvation ketoacidosis. Herein, we report of a case of starvation ketoacidosis due to long-term fasting before surgery.

**Patient concerns:**

We report of a case of metabolic acidosis due to prolonged fasting in a previously healthy 44-year-old woman during a total laparoscopic hysterectomy. Hyperventilation was observed to occur when the surgery was completed. Metabolic acidosis and hypoglycemia were demonstrated by blood gas analysis of the radial artery.

**Diagnosis:**

Metabolic acidosis.

**Interventions:**

The patient received sodium bicarbonate and 5% glucose fluid at the end of the surgery.

**Outcomes:**

The tracheal tube was successfully removed when the tidal volume of the patient returned to normal after the therapy. However, the patient suffered pulmonary edema when she was transferred to the intensive care unit (ICU). With treatments with furosemide and sodium bicarbonate, acidosis and pulmonary edema were completely corrected at 8 hours after the surgery. On the second day after the surgery, the patient suffered nausea and vomiting. Nausea and vomiting were not completely relieved on the sixth day after the operation; therefore, the patient was transferred to the Department of Gastroenterology for further therapy.

**Lessons:**

This case suggests that although the concept of enhanced recovery after surgery (ERAS) has been adopted by most physicians because of its positive outcomes, the issue of prolonged fasting still exists, and such patients may be exposed to the risk of starvation ketoacidosis.

## 1 Introduction

Enhanced recovery after surgery (ERAS) is a concept that combines various evidenced-based aspects of perioperative care to accelerate patient recovery. Initial studies on ERAS protocols that were conducted in colorectal surgery have reported a reduction in hospital stays, readmissions, and postoperative complications, in addition to improved patient satisfaction. The principles involve the interventions of preoperative, intraoperative, and postoperative periods. The most important principle during the perioperative period is fasting time.^[1]^ Traditionally, patients have been told to fast from the time point of midnight before the surgery in order to reduce the risk of pulmonary aspiration. The current opinion suggests that the intake of a high-caloric carbohydrate drink up to 2 hours before surgery reduces preoperative thirst, hunger, and anxiety in patients undergoing abdominal surgery.^[2]^ Although the concept of ERAS has been adopted by most physicians because of its positive outcomes, the principle of shortening the preoperative fasting and drinking time period is not well implemented in patients. The fasting time period can even extend to 3 days in some patients, and prolonged fasting may be the cause of starvation ketoacidosis. Herein, we report on a case of starvation ketoacidosis due to long-term fasting before surgery.

## 2 Case report

A previously healthy 44-year-old woman was admitted to the hospital for CINIII, which was indicated by the use of a cervical biopsy. Additionally, she was scheduled to undergo curettage and a total laparoscopic hysterectomy. The patient had no history of disease, drug allergies, or alcoholism. A chest radiograph, electrocardiogram, and laboratory examination revealed no abnormalities. A physical examination revealed a body temperature of 37.2 °C, a heart rate of 89 beats/min, a respiratory rate of 19 breaths/min, and blood pressure of 132/60 mmHg. The patient's height was 155 cm, and the patient's weight was 55 kg. There were no special circumstances to report before the surgery except that the patient received almost no solid foods for 3 days.

After the patient arrived in the operating room, general anesthesia was scheduled. A total of 2 mg midazolam, 120 mg propofol, 20 μg sufentanil, and 5 mg cisatracurium were administered for the induction of anesthesia. After the intubation, 3% sevoflurane was used for the maintenance of anesthesia. The initial postintubation ventilator settings involved a volume-control ventilation mode, a volume of 6 to 8 mL/kg, a respiratory rate of 12 to 15 breaths/min, a P_ET_CO_2_ of 30 to 40 mmHg, and a FiO_2_ at 100%. The patient suffered hypotension during the operation, with blood pressure being measured as low as 78 to 80/35 to 45 mmHg. After fluid therapy, blood pressure was increased to 85 to 90/45 to 50 mmHg, which persisted for nearly 30 minutes. Three hours later, the surgery was completed, and a 1500 mL crystalloid solution and a 500 mL colloid solution were administered. The intraoperative bleeding was 20 mL, and the urine output was 400 mL. The spontaneous breathing of the patient was recovered, with a tidal volume of 1120 mL, a P_ET_CO_2_ of 22 mmHg, and a respiratory rate of 8 to 10 breaths/min for 30 minutes from the end of the surgery. The patient was unconscious and unresponsive to external stimuli. Radial artery blood gas analysis was immediately performed (see Table 1). The blood glucose of the patient was 3.7 mmol/dL, and the patient was diagnosed with metabolic acidosis and hypoglycemia. Afterwards, 125 mL sodium bicarbonate was immediately administered to correct the metabolic acidosis, and 5% glucose solution was administered to raise the blood glucose level. The tidal volume of the patient slowly declined, while the P_ET_CO_2_ slowly increased. After 10 minutes, the tidal volume of the patient was 887 mL, and the P_ET_CO_2_ was 28 mmHg. The blood gas analysis indicated that the BE was declined. Approximately 10 minutes later, the patient opened her eyes with no stimulation and began to swallow. She coughed when oral suction was applied. At this time, the tidal volume of the patient was 600 mL, and the P_ET_CO_2_ was 33 mmHg. Afterwards, the tracheal tube was removed while the pulse oxygen saturation (SPO_2_) level was 95%. The patient was transferred to the ICU for further treatment, and the SPO_2_ of the patient declined to approximately 83% to 85%. An auscultation revealed crackles in both lungs, and 20 mg furosemide was immediately applied to promote diuresis, while liquid infusion was limited. At this time point, the urine volume was 1000 mL. Eight hours after the operation, the blood gas analysis demonstrated that the acidosis had been completely corrected. The patient suffered nausea and vomiting on the second day after the operation, without indications of hematemesis, abdominal pain, and distention. The abdominal ultrasound test was not eventful. At the same time, the patient complained that there were symmetrical burning sensations in the extremities without the presence of movement abnormalities, and the nerve reflex was negative. After the administration of a symptomatic treatment, the numbness and pain of the extremities were significantly reduced on the fourth day after the operation. The patient still presented with nausea and vomiting on the sixth day after the operation. Therefore, she was discharged from our hospital and was admitted to the Department of Gastroenterology in another hospital.

**Table 1:**
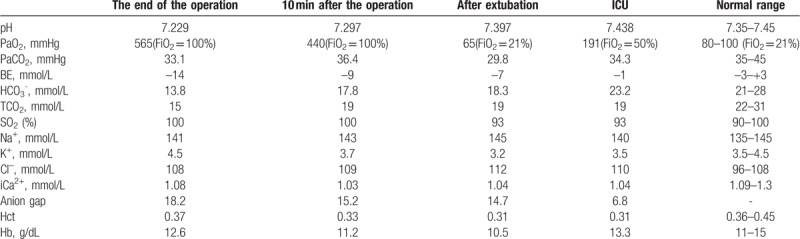
Serial arterial blood gas results.

## 3 Discussion

In this case, the condition of acidosis during the operation was considered to be starvation ketoacidosis that was caused by prolonged fasting. The main reasons for this diagnosis are as follows. Firstly, the patient denied having liver and kidney diseases, and she also denied having histories of alcoholism, dietary issues, and drug abuse. She also stated that she was not a vegetarian. The preoperative examination demonstrated that the plasma creatinine and urea nitrogen levels were normal after the operation, and there was no other disease that resulted in chronic acidosis. Second, the patient had received almost no solid food for 3 days because of the preoperative preparation before the operation, which may have resulted in insufficient sugar intake. Furthermore, during the preoperative preparation period, a routine oral administration of 1000 mL lavage fluid is needed to clean the gastrointestinal tract, which may have resulted in a massive loss of alkaline intestinal fluid and may have caused incentives for acute acidosis. Although the patient did not have their ketone body levels checked, the blood glucose level was observed to be low at the end of the operation, which was consistent with the characteristics of starvation ketoacidosis.

Clinically, metabolic acidosis is characterized by a decrease in serum HCO_3_^–^ levels, accompanied by an increased arterial partial pressure of carbon dioxide (PaCO_2_) and pH of blood. Metabolic acidosis can be divided into acute acidosis (lasting for several minutes to several days) and chronic acidosis (lasting several weeks to several years). Acute metabolic acidosis is usually associated with an excessive production of nonvolatile acidic substances, while chronic acidosis is associated with chronic kidney disease.^[3]^ The most common causes of acute metabolic acidosis are diabetic ketoacidosis and lactic acidosis. The most common cause of ketoacidosis is poor diabetes control or acute infection. Other common causes include starvation ketosis, alcoholic ketoacidosis, and an excessive use of a salicylic acid drug.^[4]^

Normally, insulin can promote glucose metabolism, which provides energy for the body. Conversely, it can inhibit the decomposition of fat. Long-term fasting reduces the source of blood glucose, and decreases in insulin levels in the body can mobilize fat decomposition; however, such fasting produces excessive ketone bodies (acetone, acetoacetic acid, and beta hydroxybutyric acid) during the process of fat decomposition, which can cause metabolic acidosis when the levels exceed the reserves of alkaloids in the body.^[5]^ After 12 to 14 hours of fasting, mild ketoacidosis may occur, although the blood pH may still be higher than 7.3.^[6]^ In nonpregnant adult volunteers, ketone production was 3 times the normal level on the third day of fasting compared with the second day of fasting.^[7]^ An observational study found that the incidence of perioperative ketonemia was 4.9%.^[8]^ Fasting may lead to severe acid–base balance disorders when a combined stress response occurs, such as a large glucose demand stimulated by the operation, and this change has been reported in pregnant women, children, and the elderly.^[9–13]^

For the treatment of starvation ketosis, the main aim is to reduce gluconeogenesis. To inhibit ketogenesis, patients can take sugar-containing liquids alone. For patients with hypoglycemia, increases in blood glucose levels can promote endogenous insulin production, and insulin can reverse ketogenesis. It has also been reported that insulin can be added to the sugar-containing liquid, but the blood glucose level should be closely monitored to prevent the occurrence of hypoglycemia, and the recommended monitoring time is 24 to 72 hours.^[14]^

Acute acidosis will cause a reduction in myocardial contractility, as well as vasodilation and an insensitivity to exogenous catecholamines, which can lead to persistent hypotension and may even cause ventricular fibrillation. It can also lead to a decrease in the production of glycerides of 2,3-DPG, which results in the reduction in the binding force of hemoglobin and oxygen; the activation of inflammatory reactions; the inhibition of the immune system, a condition of the body being more susceptible to infection; a reduction in cell productivity; and the acceleration of apoptosis. In patients who have suffered ketoacidosis, it is still controversial as to whether sodium bicarbonate is useful for correcting acidosis. In theory, the increase of blood pH increases the cellular response to insulin, but there is no evidence that an infusion of sodium bicarbonate can accelerate the recovery of patients and reduce the days of hospitalization.^[3]^ Kraut and Madias^[4]^ have observed that in patients who suffered ketoacidosis and when the blood pH <7.1, along with complications of prolonged hypotension and cardiovascular system symptoms (such as arrhythmia), the administration of insulin and fluid therapy does not work, and 5% sodium bicarbonate can be used to maintain the blood pH value above 7.2. It is worth noting that when sodium bicarbonate is used for the correction of acidic conditions, a rapid infusion of 5% sodium bicarbonate may result in volume overload and may cause a risk of acute pulmonary edema. The patient suffered severe acidosis during surgery; the tidal volume was observed to increase, and the respiratory rate decreased. After the analysis of the arterial blood gas, as well as the use of rapid rehydration and an acid correction using sodium bicarbonate, the tidal volume slowly returned to normal, consciousness was recovered, and the tracheal tube was removed. However, acute pulmonary edema occurred after the infusion of 5% sodium bicarbonate, which may be related to the excessive volume load that was caused by the rapid administration of fluid.

## 4 Conclusion

Prolonged fasting before an operation remains a practice. Prolonged fasting during the preoperative period, a large amount of alkaline intestinal fluid loss (caused by preoperative oral enema) and the effects of anesthesia and surgery will increase the levels of glucocorticoids. All of these factors will then accelerate lipid metabolism, which may cause starvation ketoacidosis. Therefore, we have to be cautious of these conditions in clinical work.

## Author contributions

**Writing – original draft:** Wenqin Zhou.

**Writing – review & editing:** LinLi Luo.

## References

[1] Ituk U Habib AS Enhanced recovery after cesarean delivery. F1000Res 2018;7:pii: F1000 Faculty Rev-513. 10.12688/f1000research.13895.1 PMC5931266 29770203

[2] Hohenberger H Delahanty K Patient-centered care-enhanced recovery after surgery and population health management. AORN J 2015;102:578–83. 26616317 10.1016/j.aorn.2015.10.016

[3] Kraut JA Madias NE Metabolic acidosis: pathophysiology, diagnosis and management. Nat Rev Nephrol 2010;6:274. 20308999 10.1038/nrneph.2010.33

[4] Kraut JA Madias NE Treatment of acute metabolic acidosis: a pathophysiologic approach. Nat Rev Nephrol 2012;8:589–601. 22945490 10.1038/nrneph.2012.186

[5] Cahill GF Jr Fuel metabolism in starvation. Annu Rev Nutr 2006;26:1–22. 16848698 10.1146/annurev.nutr.26.061505.111258

[6] Owen OE Caprio S Reichard GA Jr Ketosis of starvation: a revisit and new perspectives. Clin Endocrinol Metab 1983;12:359–79. 6347450 10.1016/s0300-595x(83)80046-2

[7] Garber AJ Menzel PH Boden G Hepatic ketogenesis and gluconeogenesis in humans. J Clin Invest 1974;54:981–9. 4430728 10.1172/JCI107839 PMC301639

[8] Ohkawa H Iwakawa T Ohtomo N Clinical study on intraoperative hyperketonemia in non-diabetic surgical patients under general anesthesia. Masui 1993;42:1803–7. 8301829

[9] Frise C Attwood B Watkinson P Life-threatening ketoacidosis in a pregnant woman with psychotic disorder. Obstet Med 2016;9:46–9. 27512491 10.1177/1753495X15621153 PMC4950438

[10] Karpate SJ Morsi H Shehmar M Euglycemic ketoacidosis in pregnancy and its management: case report and review of literature. Eur J Obstet Gynecol Reprod Biol 2013;171:386–7. 24183348 10.1016/j.ejogrb.2013.09.034

[11] Burbos N Shiner AM Morris E Severe metabolic acidosis as a consequence of acute starvation in pregnancy. Arch Gynecol Obstet 2009;279:399–400. 18592261 10.1007/s00404-008-0715-3

[12] Bai K Fu Y Liu C Pediatric non-diabetic ketoacidosis: a case-series report. BMC Pediatr 2017;17:209. 29258472 10.1186/s12887-017-0960-3 PMC5735941

[13] Iwata H Tsuzuki S Iwata M Ketoacidosis due to a Low-carbohydrate diet in an elderly woman with dementia and abnormal eating behavior. Intern Med 2017;56:2671–5. 28883241 10.2169/internalmedicine.8689-16 PMC5658538

[14] Causso C Arrieta F Hernández J Severe ketoacidosis secondary to starvation in a frutarian patient. Nutrición Hospitalaria 2010;25:1049–52. 21519781

